# The Microbial Pecking Order: Utilization of Intestinal Microbiota for Poultry Health

**DOI:** 10.3390/microorganisms7100376

**Published:** 2019-09-20

**Authors:** Joel J. Maki, Cassidy L. Klima, Matthew J. Sylte, Torey Looft

**Affiliations:** 1 Food Safety Enteric Pathogens Research Unit, National Animal Disease Center, Agricultural Research Service, United States Department of Agriculture, Ames, IA 50010, USA; joel.maki@usda.gov (J.J.M.); cassidyklima@gmail.com (C.L.K.); matthew.sylte@usda.gov (M.J.S.); 2 Interdepartmental Microbiology Graduate Program, Iowa State University, Ames, IA 50011, USA; 3 Oak Ridge Institute for Science and Education (ORISE), ARS Research Participation Program, Oak Ridge, TN 37830, USA; 4 Veterinary Diagnostic Laboratory, Iowa State University, Ames, IA 50011, USA

**Keywords:** poultry, microbiota, microbiome, antibiotics, metabolomics, alternatives to antibiotics

## Abstract

The loss of antibiotics as a tool to improve feed efficiency in poultry production has increased the urgency to understand how the microbiota interacts with animals to impact productivity and health. Modulating and harnessing microbiota-host interactions is a promising way to promote poultry health and production efficiencies without antibiotics. In poultry, the microbiome is influenced by many host and external factors including host species, age, gut compartment, diet, and environmental exposure to microbes. Because so many factors contribute to the microbiota composition, specific knowledge is needed to predict how the microbiome will respond to interventions. The effects of antibiotics on microbiomes have been well documented, with different classes of antibiotics having distinctive, specific outcomes on bacterial functions and membership. Non-antibiotic interventions, such as probiotics and prebiotics, target specific bacterial taxa or function to enhance beneficial properties of microbes in the gut. Beneficial bacteria provide a benefit by displacing pathogens and/or producing metabolites (e.g., short chain fatty acids or tryptophan metabolites) that promote poultry health by improving mucosal barrier function or immune function. Microbiota modulation has been used as a tool to reduce pathogen carriage, improve growth, and modulate the immune system. An increased understanding of how the microbiota interacts with animal hosts will improve microbiome intervention strategies to mitigate production losses without the need for antibiotics.

## 1. Introduction

The US is the largest producer of poultry worldwide, but in response to ever-increasing demands for high quality, low cost meat, the poultry industry has needed to keep pace by increasing production volumes, improving genetics and nutrition, and controlling disease [1]. Historically, antimicrobials have been a key tool used in animal production to both enhance growth and prevent and treat disease. However, concern for maintaining the efficacy of medically important drugs led to restrictions on the use of antibiotics in animal food production, limiting the off-label applications and feed efficiency uses of antibiotics important for human health [2]. As a result, emphasis has shifted to non-antibiotic methods to support production.

Intestinal health can dictate both nutrient uptake and disease status in animals, and is impacted by both the gut microbiota and host immune function. As a result, understanding how both factors influence production parameters is important to develop alternative tools that provide predictable outcomes on poultry health and growth. For example, identifying bacterial populations that are impacted by antimicrobials or feed additives can highlight ways to successfully manipulate seeding or the establishment of populations beneficial to animal growth. Additionally, strategies that enhance immune efficiency can be used to selectively inhibit potentially pathogenic populations, limiting incidences of animal diseases and foodborne illnesses. The age of birds is also a significant factor influencing gut microbiota composition, and the metabolites produced by different bacterial populations can impact the development and/or maintenance of immune cells in the intestinal tract. Understanding these processes can provide unique production strategies that maximize production and promote animal health in the absence of antibiotics.

**Figure 1:**
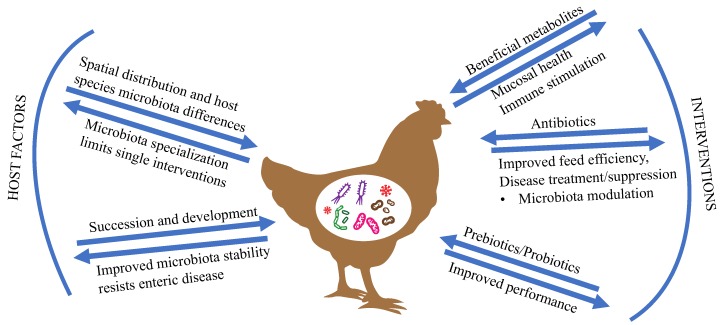
Factors influencing the poultry microbiota composition and potential outcomes that influence animal health. Microbiome factors discussed in the review that influence the composition and/or functions of the microbiota are indicated with arrows pointing inward. Outward pointing arrows suggest potential or desired outcomes to the factors.

In this review, we will examine factors known to influence the poultry microbiota composition, promising microbiota modulation intervention strategies and bacterial-derived metabolites to improve poultry health and production. Targeting intestinal health as a way to improve performance is not new. However, strategies that do not rely on antibiotics require targeted intervention and often need to be used in combination (vaccination, probiotics, prebiotics, biosecurity) to improve both feed efficiency and control infections [1]. Microbiome interventions often target specific members or functions of the microbiota, and understanding the outcomes on microbial populations and the host are essential in predicting successful intervention. Prediction requires particular understanding of the microbes that contribute to disease, support proper gut development, interact with the gut microbiota to improve nutrient absorption, and either support or suppress the avian immune system. An overview of host and environmental factors that influence the intestinal microbiota, as well as common microbiota interventions and possible outcomes to poultry health, are summarized in Figure 1. Preventing colonization of pathogens and controlling microbial succession in developing birds is a means for maintaining flock health. Key to modulating the microbiota is understanding how early bacterial colonizers are acquired and develop into a stable, beneficial microbiota.

## 2. Microbial Spatial Diversity

**Figure 2:**
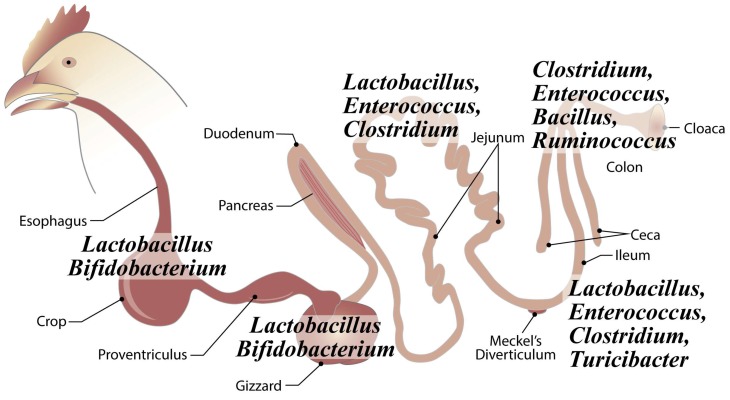
Chicken intestinal communities differentiate along the intestinal tract. The most abundant bacterial taxa are indicated for each gut compartment.

The chicken gastro-intestinal tract (GIT) includes compartments with varied physiological roles and environments that drive a spatial distribution of microbial populations. The GIT serves as the home for anywhere between 500–1000 bacterial species, comprising up to 100 trillion cells in total [3,4]. In mature birds, *Lactobacillus* is the dominant genus in the crop and gizzard, but *Bifidobacterium* may also be present (Figure 2) [5]. The duodenum and jejunum are colonized at low densities, in part, due to high bile acid concentrations and low pH, but *Lactobacilli, Enterococci,* and *Clostridiaceae* are commonly detected (Figure 2) [5,6,7,8]. The ileum is the terminal segment of the small intestine and has the greatest microbial density and diversity of the small intestine, where *Lactobacillus, Enterococcus*, *Clostridium*, and *Turicibacter* are found in high abundance among other genera (Figure 2) [7,9,10,11,12,13]. In the ceca, the bacterial community peaks in complexity and density, with strict anaerobes from the phylum Firmicutes, composed of the genera *Clostridium*, *Enterococcus*, *Bacillus*, and *Ruminococcus,* being found in high abundance (Figure 2) [11,14,15,16].

## 3. Microbiota Succession and Its Role in Disease

Successional patterns and mature community compositions are important for bird health, with increased microbiota diversity associated with reduced rates of enteric diseases in poultry [17]. For example, exposing chicks to the mature microbiota of adult birds increases the speed of microbial succession in the gut, resulting in the establishment of a mature microbiota at a younger age [18]. While there are health benefits from increased diversity, the presence of individual microbes is also associated with specific health outcomes [17]. *Lactococcus* colonization of the ceca promotes weight gain in chicks, while the presence of *Akkermansia* and *Prevotella* are negatively correlated with weight gain [15]. Other studies associate specific genera such as *Lactobacillus*, *Ruminococcus*, and *Clostridium* clusters IV and XIVa with enhanced bird performance [19,20,21]. As a result, understanding the microbial succession in a healthy avian gut and how production practices impact this process is important if alternative intervention strategies for disease are to be examined.

In most vertebrate species, maternal feces serve as a major route for the transmission of beneficial commensal microbes to offspring [22,23]. However, in commercial poultry production systems, the linkage between hen and chick is severed as eggs are collected from layer flocks and incubated and hatched at separate facilities [24]. Because direct vertical transmission from the hen is prevented, the microbial inoculum for chicks is limited to eggshell, litter, feed, and water [20,25,26]. Eggs are exposed to *Lactobacillus*, *Psuedomonas*, and *Bacteroides* that are present in the hen reproductive tract [27], as well as intestinal microbes during passage through the cloaca. Internalization and deposition of bacteria on the eggshell has been observed [27,28,29,30], but to colonize newly hatched chicks, the microbiota on the eggshell surface must survive the incubation period (21 days for chickens) in a highly oxygenated, nutrient-poor environment. Consequently, spore-forming bacteria are well adapted to surviving the oxygen and desiccation stress of the eggshell [31]. Firmicutes compose over 50% of the bacteria on the surface of chicken eggs, including members of the genera *Clostridia*, *Ruminococcus*, and *Lachnospiraceae*. Members of the phyla Fusobacteria, Bacteroidetes, and Proteobacteria compose >10% of the bacteria on the eggshell, including potential pathogens like *Pseudomonas* and *Salmonella* [32].

Post-hatch, the intestinal communities of chicks are highly dynamic, making the immature microbiota susceptible to interventions that can have lasting effects on immune development and host energy harvest [33]. Growing chicks have periods marked with developmental changes within the gut microbiome, characterized by dynamic changes early in succession before the eventual establishment of a more stable and complex community structure [16,17,33,34]. Around one to three days post-hatch, rapidly colonizing facultative anaerobes, including *Streptococcus* and *Escherichia,* establish in the gut, driving down the redox potential. Anaerobic bacteria of the Firmicutes phylum, including *Ruminococcus* and *Lachnospiraceae*, eventually displace these rapid colonizers. By day 10, slower-growing anaerobes like *Romboutsia* spp. become detectible, signaling a shift toward a more diverse and evenly distributed microbiota [34]. Age appears to be the greatest driver in determining chicken GIT bacterial composition, with the passage of time coinciding with increased microbiota complexity [17].

A well-functioning intestinal microbiota provides numerous benefits for the host. A rich, highly complex gut microbiota competes with potential pathogens for colonization, aiding in the exclusion of disease-causing organisms such as *Clostridium perfringens* [35,36,37,38]. The gut microbiota also stimulates the development of the poultry mucosal epithelia and intestinal immune system, providing the host with another layer of defense against potential pathogens [3,39,40,41]. Delays in microbiota succession diminish the benefits of the commensal gut microbes, making chicks especially vulnerable to enteric diseases like necrotic enteritis [42,43,44]. Imbalance within the intestinal microbiota populations, a state known as dysbiosis, is associated with inflammation and impaired digestive and immune function, often leading to production losses [39].

## 4. Poultry Species Differences

While poultry microbiotas are similar, differences exist between bird species and breeds. For example, layer chickens harbor a more complex fecal microbiota compared to broiler chickens, although this association is likely related to the longevity of layers [45]. For the most part, broilers and layers are fairly similar in regard to their successional patterns and mature community compositions, though broilers have Firmicutes as the most dominant phyla post-hatch, while Proteobacteria dominate the layer microbiota for the first seven days post-hatch, after which point Firmicutes replace the Proteobacteria as the dominant phyla [46]. Direct comparisons between layers and broilers are challenging because of the different production practices for each. This can be a challenge when comparing different chicken breeds as well, although a recent comparison between three different breeds identified >94% of the bacterial genera to be shared across the microbiotas [46]. The microbiota composition and successional pattern in turkeys is fairly similar to that of chickens, with succession and bird development taking longer in the former. In turkeys, Firmicutes increase in abundance with time, while the proportion of Proteobacteria decrease. *Lactobacilli* make up a large portion of the turkey ileum community [14]. While *Clostridium*, *Ruminococcus*, and *Lactobacillus* are prevalent in the GIT of both chickens and turkeys, the two only share ~16% similarity at the species level, suggesting a significant degree of host specificity [15]. In ducks, Proteobacteria and Firmicutes dominate the small intestine (duodenum and ileum) while Bacteroidetes dominate the ceca, differentiating ducks from both turkeys and chickens [47].

Variability in the GIT microbiota is well documented and factors such as genotype, species, bedding material, diet, and sex contribute to differences in composition [15,19,48,49,50]. Even when these factors are controlled for, individual bird-to-bird variation in the GIT microbiota exists, potentially arising from the inherent nature of modern poultry production practices [9,20,51,52]. One of the key takeaways from these studies is that the first microbes entering the intestinal tract of newly-hatched chicks can have a profound impact on how the microbiota, and the bird itself, develops, highlighting the importance of early intervention as a way to increase bird health and performance in the poultry industry, especially under the rapid turnaround times that define modern production cycles. Understanding what the target microbiota composition is when intervention decisions are made will improve the outcome predictions for the host.

## 5. Modulation of the Microbiota

In-feed additives are an important tool used in animal food production to enhance performance and improve poultry health. Many of these additives modulate the gut microbial community in ways that result in enhanced immune health, inhibition of pathogenic organisms, and/or enhancement of nutrient availability and uptake in the gut. Multiple categories of in-feed additives exist, including antimicrobials, heavy metals, probiotics, prebiotics, cocccidiostats, organic acids, vitamin and mineral oils, enzymes, and others [53]. Of these, antibiotics are a primary tool used to support the economic sustainability of intensive livestock operations, both through improving feed efficiency and maintaining animal health by preventing and/or treating disease.

Concerns over the use of antimicrobials in animal production center on the risk of associated development and the spread of antimicrobial resistance, particularly where it threatens the efficacy of therapeutics important for human health. In 2017, the US Food and Drug Administration (FDA) enacted the Veterinary Feed Directive (VFD), which prohibits the use of medically important antimicrobials for animal production purposes (growth promotion and feed efficiency) and brings their therapeutic use under the supervision of licensed veterinarians [2]. This ban does not, however, prohibit the use of non-medically important antimicrobials in-feed and the continued availability of medically important drugs for prophylactic use on the herd or flock still presents concern over prudent uses of these drugs in the US industry. In 2017, of the total drugs sold for use in animal agriculture in the US, 51% were considered medically important, with tetracyclines and penicillins accounting for 32% and 6% of the overall drugs sold, respectively [54]. Unlike the swine and beef industries, drug sales in poultry production are scarcely reported. Some of the antibiotics currently available for use in chickens in the US include bacitracin, avilamycin, monensin, lasalocid, salinomycin, tetracyclines, ceftiofur, lincomycin, virginiamycin, erythromycin, gentamicin, spectinomycin, neomycin, novobiocin, sulfa drugs, and tylosin. Of these drugs, bacitracin and the ionophore class, represented by monensin, lasalocid, and salinomycin, are not considered relevant to human health.

Antibiotics can be used in-feed throughout the production period or sporadically as therapeutics and, as a result, have the potential to impact the bird microbiota throughout its lifetime. Examining how antimicrobials directly impact the gut microbiota in poultry may help to both identify the modes of action that result in enhanced gain and highlight the relationship between these drugs and bird health and disease [55]. The use of antimicrobials affects the gut microbiota, immune response, and performance [40,56,57,58,59,60] to benefit animal health, in part, by modulating the immune system and modifying the microbiota of the gastrointestinal tract, resulting in a reduction of the total bacterial load and suppression of pathogens [61]. The direct mode of action for antibiotics on bacterial populations or growth promotion is not clearly defined and may not be consistent across products or applications. However, the age of birds has a larger effect on gut maturation than drug use [14], and antimicrobials have a greater effect on rare species than abundant ones [57]. Overall, the use of antibiotics can induce significant changes in membership, but typically does not alter the functionality of the microbiota.

Although it is believed that subtherapeutic antimicrobials stabilize the gut microbiota, improve performance, and prevent various intestinal pathologies [53], the effectiveness of antimicrobials as growth promoters may be lower than was first proposed over 50 years ago. The use of subtherapeutic antibiotics may not work to support animal performance through effectively altering any one species or gut diversity, but may suppress the overall richness of gut populations [57]. Denmark banned the use of antimicrobial growth promoters without a negative effect on mortality or performance in swine and poultry [62]. Thus, it may be possible for poultry industries to cost-effectively optimize production, without using antibiotics as growth promoters [63].

The effects of different commonly used antibiotics on the cecal microbiota of poultry are summarized in Table 1.

Antimicrobial use in poultry can have varied effects on microbial populations relevant for human disease. Enramycin is a lipopeptide that shares a similar cyclic peptide structure and activity against Gram-positive bacteria with its analog ramoplanin, a drug of importance in treating humans with multi-drug resistant infections such as methicillin-resistant *Staphylococcus aureus* and vancomycin-resistant *Enterococcus* [66]. Although resistance to ramoplanin may develop in poultry and spread to humans, the use of enramycin is not prevented in poultry. The extent to which antimicrobial resistance develops may be determined by multiple factors including the drug’s spectrum of activity, its use at either therapeutic or subtherapeutic levels, the existence of antimicrobial resistance prior to use, and the varied potential of specific drugs to promote horizontal gene transfer from commensal to pathogenic populations. Therapeutic use of tetracycline in layers increases *Enterococcus* and *Escherichia* shedding in the feces [67]. However, subtherapeutic virginiamycin use has been shown to decrease *Salmonella* and *E. coli* in the digestive tract of broilers but increase *Lactobacillus* and *Enterococcus* [68]. Early administration of amoxicillin for a period of 24h in Cobb chicks resulted in a lower abundance of *Lactobacillaceae* and higher abundance of *Enterococcaceae* in the jejunum [40].

The use of antimicrobials may impact poultry health through modulation of the immune function. For example, early administration of amoxicillin for a period of 24 h in Cobb chicks has a significant effect on the intestinal host-gene expression profile, including downregulation of immune-related genes and an upregulation of genes linked to cell development and intestinal barrier function, resulting in a significantly reduced number of macrophages in intestinal mucosal tissue [40]. Administration of enrofloxacin or amoxicillin in drinking water initially affected expression of pro-inflammatory cytokines in intestinal tissue, but the effect was temporary and did not persist [69]. Similarly, in-feed bacitracin methylene disalicylate significantly affected expression of cytokines and host-defense peptide expression in ileum and cecum of broilers [70]. These studies highlight that, although some antimicrobials may have few measurable impacts on the microbiota, drug use may significantly impact immune competence, leading to increased risk for disease development in the animal during early life. In addition, a recent study examining the use of chlortetracycline and salinomycin in broilers, the latter drug being an ionophore similar in activity to monensin, showed delayed maturation of the microbiota in response to drug exposure [71]. This delayed maturation was also paralleled with delayed development of immunity, a phenomenon that is believed to compromise gut defense function and negatively impact bird health. Although growth promotion in response to antimicrobials was observed in this study, the authors argue this was likely due to suppression of gut bacteria that, in turn, freed up nutrients for uptake, inhibition of organisms that may contribute to gastrointestinal infections, and/or suppression of host immune responses that may cause biological damage to the animal [71]. Their ultimate argument is that alternative tools, such as probiotics, can enhance gut immune function and support early gut maturation, resulting in gains similar to those achieved through antimicrobial growth promoters without negatively affecting the host immune development [71].

### Other Novel Feed Additives

Other novel feed additives are also being investigated for their growth enhancing and microbial modulation properties, including clay, heavy metals, and organic acids such as butyrate [72,73]. An investigation into the addition of biochar, bentonite, or zeolite in the feed of laying hens on the carriage of pathogens in the gut microbiota showed that, although no effects were observed in overall community richness and diversity, there was a reduction in the abundance of Proteobacteria in response to bentonite use, specifically *Campylobacter* and *Helicobacter* [74]. Another recent study examined the use of selenium nanoparticles to inhibit pathogen colonization and discovered that an intermediate concentration (0.9 mg/kg) increased the abundance of *Lactobacillus* and *Faecalibacterium*, both considered beneficial to gut health, as well as *Turicibacter* and *Staphylococcus*, both potentially pathogenic bacteria, in the cecal contents of broilers [75].

## 6. Prebiotics and Probiotics

### 6.1. Probiotics

The intestinal microbiota is vital to gut development, mucosal immunity, and the digestion of feed and nutrient absorption by the host [13]. Thus, understanding the attributes of a highly productive microbiota may aid in the development of alternatives to growth promoting antibiotics [64]. Probiotics and prebiotics are tools being explored to help reduce the dependency on antimicrobials in production [13]. Probiotics are viable bacteria that provide health benefits after ingestion, including enhancing the function of the intestinal barrier of the host, excluding potential pathogens, and maintaining homeostasis in the GIT [76]. Probiotics may benefit the host directly without microbiota-wide changes [76]. In male broilers, in-feed administration of *Bacillus subtilis* CGMCC 1.1086 resulted in higher weight gain and improved feed conversion ratio (FCR) [77]. Another study involved feeding the probiotic *Lactobacillus planatarum*, resulting in enhanced immunity, including increased thymus size along with increased serum IgG and secretory IgA [71]. Two strains commonly used as probiotics in poultry are *Lactobacillus* and *Enterococcus* spp. because both are found naturally in high concentrations within the bird GIT [72,78]. *Lactobacillus* spp. have been associated with increased body weight, enhanced goblet cell counts, and decreased *E. coli* colonization in the digestive tract among other positive health outcomes for poultry flocks [79,80,81]. Dietary supplementation of *Enterococcus* spp. increased feed conversion ratio (FCR) and broiler growth [82].

To identify novel probiotic species, a comparison of cecal microbiota differences between the best and poorest performing birds was done using the performance measures of: FCR, utilization of energy from the feed measured as apparent metabolizable energy, average daily gain, and feed intake [83]. The study identified potential members of *Lachnospiraceae, Ruminococcaceae*, and *Erysipelotrichaceae* significantly correlated with good FCR performance and some *Lactobacillus* spp. that correlated with poor performance [83]. Similar associations with poor animal performance and *Lactobacillus* spp. were reported in another study [84]. Incidentally, both studies identified *Clostridium lactatifermentans* as a potential probiotic for future development.

### 6.2. Prebiotics

Prebiotics are feed additives that suppress pathogen loads while maintaining productivity by directly feeding beneficial populations within the microbiota [85]. Commonly used prebiotics in poultry include dietary fibers such as fructooligosaccharides (FOS) and xylooligosaccharides (XOS), both of which have been reviewed more in depth in other publications [78,86]. The exact mechanisms through which prebiotics function vary depending on the type of dietary fiber and many of these mechanisms have yet to be fully elucidated. Several studies involving FOS supplementation saw increases in the *Lactobacillus* and *Bifidobacterium* populations in the ileum and cecum of broilers accompanying decreased levels of *C. perfringens* and *E. coli* while those involving XOS supplementation identified increased *Lactobacillus* and *Clostridium* cluster XIVa levels in the colon and ceca of broilers [78,87,88,89,90]. Many of the microbial community modulatory effects and subsequent health benefits of FOS and XOS can be attributed to their fermentation by beneficial commensals, such as *Bifidobacterium*, into short chain fatty acids (SCFAs) [91,92]. The benefits of these SCFAs will be explored in greater detail later in this article.

## 7. BA-Modification and Modulation

While the exact mechanisms through which antimicrobials function to increase weight gain and other performance metrics in livestock species have yet to be fully elucidated, antibiotics may inhibit bile acid (BA)-modifying populations in the gut, leading to the enhancement of lipid absorption and overall energy harvest by the host [58,93,94]. This observation provides researchers with a potential target population for the development of interventions that mimic the effects of antimicrobial growth promoters without the associated drawbacks of antimicrobial resistance development and dissemination.

**Figure 3:**
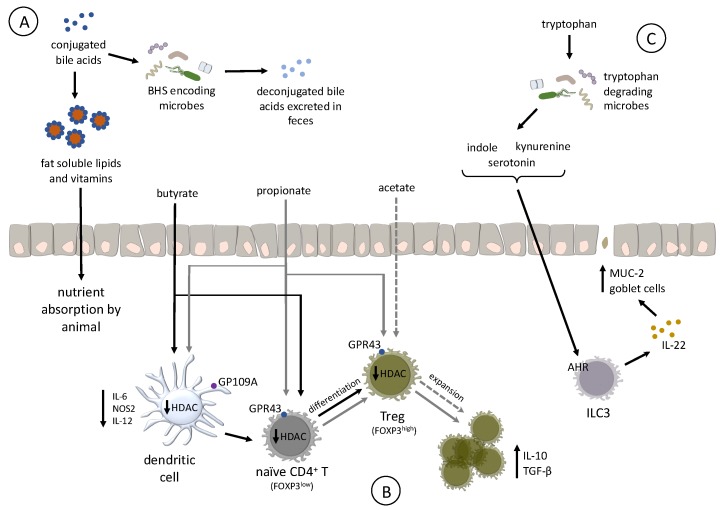
Microbial functions have many effects within the intestinal tract. (**A**) Bile acids (BA) emulsify lipids and vitamins, improving absorption by the host. Bile salt hydrolases produced by members of the microbiota deconjugate BAs, reducing their solubility and increase their excretion in the feces. Loss of BAs inhibits nutrient absorption and reduces BA recycling. (**B**) Short chain fatty acids (SCFA) are bacterial fermentation products that can be an energy source for colonocytes and impact immune cell development. Butyrate and propionate have been shown to inhibit nuclear histone deacetylases (HDAC) in macrophage and CD4 cells, prompting the generation of regulatory T cells (Treg). These Treg cells modulate the immune system, maintaining self-tolerance; suppression of allergy-, asthma- and pathogen-induced immunopathology; etc. Each unique arrow (back, gray, or dashed) from each SCFA, shows the respective downstream effects of each SCFA. (**C**) Tryptophan can be degraded by microbes into a variety of intermediates including indole and serotonin. These molecules are endogenous ligands for the aryl hydrocarbon receptor (AHR) that is present on multiple adaptive and innate immune cells. When AHR is signaled in interepithelial type 3 innate lymphoid cells, these cells produce IL-22. This cytoprotective cytokine supports the acts to strengthen epithelial barrier functions by inducing the secretion of antimicrobial peptides from epithelial cells, production of mucins (MUC-2), and proliferation of intestinal goblet cells.

Bile acids are synthesized in the liver from cholesterol compounds, conjugated with taurine to increase their solubility, and secreted from the gallbladder into the duodenum upon feeding [95,96,97]. Once in the intestine, BAs emulsify poorly soluble lipids and vitamins, enhancing their absorption by the host [98]. However, modifications to the BA pool by microbes that encode bile salt hydrolase (BSH) reduces BA solubilization ability and, subsequently, nutrient absorption [99]. BA deconjugation also results in high levels of its excretion in the feces, increasing energy expenditure on the part of the animal to synthesize more BA, translating into reduced growth rate (Figure 3A) [100,101].

Different classes of antibiotics have the common effect of disrupting and reducing the BA-modifying microbes in the intestinal tract of poultry and other livestock species [58,94,102]. In these studies, decreases in BSH-encoding organisms, like *Lactobacillus salivarius*, led to increases in bird performance. Identifying BSH inhibitors, such as riboflavin and zinc, may be of use for in-feed supplementation to prevent the deconjugation of taurine from BAs and increase lipid absorption in the diet. However, more needs to be done to identify BSH inhibitors, determine their mechanisms of action, and assess the impacts these inhibitors have on both the host and the microbiota [103]. The use of BSH-encoding organisms, such as *Lactobacilli* and *Enterococci*, as probiotics should be questioned because they may negatively impact dietary lipid absorption and bird performance [59].

## 8. Bacterial Metabolite Interactions with Host

The metabolome is a combination of molecules *de novo* produced or modified by microbes and the host. As a diverse microbial ecosystem, the intestinal microbiome produces many bioactive metabolites that act locally at the microbial-host interface to promote homeostasis and development of intestinal tissues [104], as well as acting extra-intestinally in organs such as the brain or spleen [105,106]. In poultry, strategies that affect the composition of the intestinal microbiota (antibiotics, probiotics, and prebiotics) may impact the functional metabolome [107,108], and ultimately affect gut health and performance. A complex, mutualistic interplay between the intestinal microbiome, epithelium, and immune cells is vital for gastrointestinal homeostasis [109]. Although an understanding of how microbial metabolites shape the intestinal immune development is incomplete, several classes of molecules, including bacterial-derived short chain fatty acids (SCFAs) and tryptophan metabolites, are well characterized as modulators of host immune development and intestinal homeostasis. Most of these data were obtained from rodent models or human clinical samples with different intestinal pathologies. How SCFAs or tryptophan metabolites affect immune development in poultry is mostly unknown, but represents an exciting new field of study to impact immune development in an era where fewer therapeutic options are available to treat mucosal infectious diseases in poultry.

## 9. Bacterial-Derived SCFAs and Host Immune Development

Microbial-derived SCFAs are a diverse group of molecules one to six carbons in length that benefit the host by providing energy to the intestinal epithelium, produce tolerance to microbial-associated microbial patterns (MAMPs) in intestinal mucosa [110], and are protective against intestinal immunopathology such as inflammatory bowel disease (IBD) [111,112]. Acetate (C2), proprionate (C3), and butyrate (C4) are produced in the highest quantities and are the best studied [113,114], but additional SCFAs including lactate, succinate, valerate, and others may also benefit gut health.

A comprehensive list of SCFA-producing bacteria in vivo is currently incomplete, but is an ongoing field of research [115,116] that is complicated by multiple different bacteria that are requisite to produce SCFA precursors. In humans, for example, acetate dependency on butyrate production by *Faecalibacterium prausnitzii* was demonstrated in vitro and in vivo [117,118]. Lactate, a less abundant SCFA produced by many bacteria in the human intestinal tract, may act as a substrate for production of proprionate and butyrate by *Coprococcus catus* [119] or just butyrate by *Anaerostipes caccae* or *Eubacterium hallii* [120]. Some individual bacterial species isolated from the chicken ceca are capable of producing butyrate [121,122], but less is known about the identity of other SCFA-producing bacteria or their required precursors in poultry [123].

Many SCFA-producing bacteria are mucosal associated and generate high concentrations of SCFAs in close proximity to host intestinal epithelium [124]. As an energy source, the SCFAs proprionate and butyrate can enter host cells via active transporters such as sodium-coupled monocarboxylate transporter 1 [125,126]. Not all absorbed SCFAs reach systemic circulation, and instead act locally in the intestinal tract or liver. Butyrate, the major energy source for colonocytes, is locally metabolized after being transported to the epithelial cells [127,128]. In mammals, butyrate that escapes beyond the intestinal tract is metabolized in the liver [129,130]. The majority of acetate and propionate are not metabolized by the intestinal mucosa and enter the liver, where propionate is metabolized, but acetate enters into peripheral circulation [129].

Host cells respond to SCFAs as both extracellular and intracellular signaling molecules. Intestinal epithelial and immune cells such as dendritic cells and macrophages express SCFA receptors and, as extracellular ligands, SCFAs are agonists for different G-protein-coupled receptors such as free fatty acid receptor 2 (FFAR2, also known as GPR43) and free fatty acid receptor 3 (FFAR3 or GPR41) [131,132,133] and the hydroxycarboxylic acid receptor 2 (HCAR2 or GPR109A) [134]. These receptors differentially bind and transduce signals from different SCFAs.

Production of specific SCFAs supports the development of immunological tolerance by affecting the expansion or differentiation and development of regulatory T cells (Tregs) in the intestinal lamina propria [111]. Separated by a single cell layer of epithelium, Tregs block effector T cells and resident antigen presenting cells in the intestinal tract to become activated by the high concentration of MAMPs found in the intestinal lumen. As a result, mammalian Tregs promote intestinal homeostasis. In mammals, these cells are characterized by surface expression of CD4, and high amounts of CD25 (IL-2 receptor chain alpha), transcription factor Forkhead box P3 (FOXP3), as well as the production of the tolerogenic cytokine IL-10 (Figure 3B) [135,136]. It is unclear whether microbial-produced SCFAs affect the differentiation or expansion of Tregs in poultry, mainly because FOXP3 is not currently annotated in the genomes of either turkeys or chickens. Recent RNA-Seq analysis suggests that some birds (*Parus humilis, Falco peregrinus*, and *F. cherrug*) express *FOXP3*, but the gene is missing from poultry genomes due to a sequencing artifact [137]. At this time, there are no data to support the hypothesis that bacterial SCFAs affect Treg expansion or differentiation in poultry, but butyrate in poultry appears to share additional functions characterized in humans. For example, butyrate treatment of the human colonic epithelial cell line HT-29 strongly induced expression of human beta defensins-1 and -2 [138]. Ex-vivo treatment of chicken cecal tissue with butyrate increased expression of host defense peptides (HDP) beta-defensins and cathelicidin B1 [139]. Chickens fed butyrate significantly reduced cecal colonization by *Salmonella* [139,140], which may be due to increased cecal HDP expression. These data suggest that butyrate in poultry has an immunomodulatory property to confer resistance to some infectious diseases and may affect Treg expansion and differentiation.

Differentiation of naïve CD4^+^ T cells to Tregs depends on inhibition of histone deacetylase (HDAC) in both dendritic cells and T cells [111,141]. In humans and mice, propionate and butyrate inhibit HDCA by acting as intracellular signaling molecules and broadly affect transcriptional regulation by inhibiting HDAC, and promote activation of histone acetyltransferases [128,142]. Butyrate is the most-potent HDAC inhibitor, targeting classes I and III [143], whereas propionate is a less potent and defined HDAC inhibitor. Butyrate administered orally to chickens induced hyperacetylation of histones, suggesting it functions as an HDAC [144]. While acetate and propionate, but not butyrate, stimulate expansion of existing Tregs in the colon of mice, proprionate and butyrate, but not acetate, enhancing the differentiation of naïve CD4^+^ T cell to Tregs. Although germ-free mice have colonic Tregs, their abundance is markedly reduced due to the absence of acetate and proprionate, but can be rescued by dietary supplementation with acetate or propionate [112]. Separately, butyrate enhanced differentiation of Tregs in humans by creating an anti-inflammatory phenotype in macrophages and dendritic cells through GP109A signaling [145]. Based on the existing research in humans and mice, it is possible that identifying prebiotics that favor growth of SCFA precursor-producing bacteria, or probiotics that produce SCFAs in the poultry gut may be valuable tools to promote alternative strategies to support gut health and resistance to some infectious diseases.

## 10. Bacterial-Derived Tryptophan Metabolites and Host Immune Development

Tryptophan is an essential amino acid and must be supplied through the diet to meet the host’s nutritional needs. Bacteria in the intestinal tract can degrade dietary tryptophan to a variety of intermediates including indole [146], serotonin, or kynurenine, of which some are endogenous ligands for the aryl hydrocarbon receptor (AHR) [147]. The AHR is a transcription factor ubiquitously expressed in mammalian cells and was originally characterized as a cellular response to toxic xenobiotics such as halogenated polycyclic aromatic hydrocarbons [148]. In mammals, signaling of AHR with bacterial-derived endogenous ligands is vital to promote intestinal homeostasis [149] and immune development (Figure 3C) [150]. In mice, signaling via AHR in intraepithelial type 3 innate lymphoid cells (ILC3s) [151] maintains production of the cytoprotective cytokine IL-22 [152,153], and protects against some forms of intestinal pathology [147] by inducing the secretion of antimicrobial peptides from epithelial cells, production of mucins (MUC-2), and proliferation of intestinal goblet cells [154]. Mice deficient in AHR demonstrate multiple immunological deficits, including reduced resistance to infection with the bacteria *Listeria monocytogenes* or *Citrobacter rodentium,* as well as an exaggerated immunopathological response to dextran sodium sulfate (DSS)-induced colitis [106,147,152,153,155,156]. The role ILC3s play in promoting intestinal homeostasis in poultry is unknown because they are not yet characterized in chickens or turkeys. However, oral treatment with endogenous AHR ligand 3,3′-diindolylmethane was efficacious to reduce parasite-induced intestinal inflammation in chickens [157], indicating an AHR-induced cytoprotective mechanism which is likely to also exist in poultry.

Only a few commensal intestinal bacteria (*Peptostreptococcus russellii* [158] and *Lactobacillus* spp. [147,159]) are known to produce endogenous AHR ligands. In the human intestinal microbiome, *Clostridium sporogenes* decarboxylates tryptophan leading to the production of the neurotransmitter tryptamine [160], as well as the production of indoleacetic acid and indolepropionic acid, which are known to affect intestinal permeability and host immunity [159,161,162]. Bacteria capable of producing tryptophan metabolites in poultry are mostly unknown. An avian pathogenic *E. coli* isolate possessed the tryptophanase *tnaA* [163], but functionality was not demonstrated. Tryptophanase, which deaminates tryptophan to form indole, is expressed by the commensals *E. coli* and *Lactobacillus* spp. [146,164]. As mentioned previously, these genera are abundant in the poultry GIT microbiota. It is possible that these, or other bacteria are involved in production of indole, a key precursor of several endogenous AHR ligands that affect immune development. We recently demonstrated that feeding bacitracin to turkeys affected the concentration of indole metabolites in cecal contents, some of which may activate AHR [108]. The use of specific prebiotics and probiotics to affect tryptophan metabolism may be vital tools to promote gut health, disease resistance, and immune development to enhance poultry health.

## 11. Current Limitations of Microbiota Studies

Of the studies discussed here, the majority profiled the microbiota using 16S rRNA gene amplicon sequencing. Although this method is cost effective for mapping large-scale shifts in the microbiome, many studies now highlight that key responses to in-feed additive use occur at the species level. Because 16S rRNA gene amplicon sequencing resolves to the family or genera level, its application to identify at a species level resolution is limited. Metagenomic sequencing, although able to resolve the microbiome to the species or strain level, is still largely cost prohibitive and the dependency on short read sequencing to obtain coverage in complex samples provides challenges for assemblies. As technologies continue to evolve and become more affordable, it is likely that sequencing of the metagenome will help to better discriminate the rare species and strains that are most affected by in-feed additives and provide information that will aid in both our understanding and the effective manipulation of microbial gut communities.

## 12. Conclusions

The microbiota can be viewed as a collection of microbial species or taxa present, as well as their collective functions. These communities provide benefits for poultry production, such as stimulation of the immune system, pathogen displacement, and improving nutrient absorption. While antibiotics have been a powerful tool used in animal agriculture for decades, concern over antibiotic resistance has led to an urgent need to understand how animal microbiomes affect animal health. Factors such as age, gut compartment, and health status influence the microbiota composition, but similar trends exist across poultry species that can be exploited to improve intestinal health, and performance. Good interventions target key functions to improve intestinal health or specific taxa responsible for performance losses or disease, but promising new interventions target specific functions of beneficial bacteria such as production of SCFA or tryptophan metabolites to stimulate cellular pathways with systemic effects on poultry health. Future targeted approaches in poultry production require deep understanding of how the microbiome influences bird health and production.

## Figures and Tables

**Table 1:** Antibiotics used in poultry production, and their impact on the cecal microbiota.

Antibiotic	Species	Changes in Relative Abundance of Cecal Microbiota	Reference(s)
Bacitracin zinc	Broiler chicken	Decrease in *Lactobacillus* and *Eubacteria* Increase in *Clostridiales* (*Faecalibacterium* and *Ruminococcus*)	[64]
Avilamycin	Broiler chicken	Decrease in *Lactobacillus* and *Clostridiales*	[57,64,65]
Virginiamycin and monensin	Broiler chicken	Decrease in Firmicutes Increase in Gammaproteobacteria (*E. coli*)	[14]
Monensin	Broiler chicken	Decrease in *Erysipelotrichaceae*, *Lactobacillaceae*, *Enterococcaceae* and *Insertae Sedis* XIV Depleted *Roseburia, Lactobabcillus,* and *Enterococcus*	[14]
Chlortetracycline	Broiler chicken	Decrease in Gammaproteobacteria (*E. coli*) Increase in *Bifidiobacterium*	[60]
Enramycin	Broiler chicken	Decrease in Firmicutes, *Clostridium* XI and unclassified *Peptostreptococcaceae* Increase in *Clostridium* XIVb and *Anaerosporobacter*	[57,65]
Tylosin	Broiler chicken	Decrease in *Roseburia* Increase in *Escherichia* and *Hespellia*	[14]
